# Whole-Genome Sequencing of Neisseria gonorrhoeae Isolates with Antimicrobial Resistance Plasmids from a Homeless Community in Colombia

**DOI:** 10.1128/mra.00623-22

**Published:** 2022-10-17

**Authors:** Oscar Mauricio Gómez, Diego E. Velez, Juan G. McEwen, Aracelly Villegas

**Affiliations:** a School of Microbiology, University of Antioquia, Medellín, Colombia; b Cellular and Molecular Biology Unit, Corporación para Investigaciones Biológicas, Medellín, Colombia; c Department of Microbiology and Parasitology, School of Medicine, University of Antioquia, Medellín, Colombia; University of Maryland School of Medicine

## Abstract

Neisseria gonorrhoeae is a pathogenic bacterium causing sexually transmitted infections, and it is associated with high antibiotic resistance rates. Here, we describe the genome sequences of four isolates from a homeless community in Colombia.

## ANNOUNCEMENT

Neisseria gonorrhoeae is a Gram-negative diplococcus that colonizes only humans (1). It infects the urethra, the endocervix, and occasionally the ocular, nasopharyngeal, and rectal mucosa (2). Recently, the WHO, using whole-genome sequencing (WGS) in conjunction with linked epidemiological and phenotypic data (including susceptibility profiles), described several gonococcal genotypes in European countries (3), some of them associated with antimicrobial resistance (AMR) (4). The WGS of N. gonorrhoeae isolates has been proposed as a cost-effective tool for screening for AMR in this pathogen (4–6). However, the understanding of the molecular epidemiology of N. gonorrhoeae strains from developing countries such as Colombia is limited. Our objective was to use WGS to describe the genomic characteristics of N. gonorrhoeae isolates from a homeless community in Colombia.

Among homeless participants with urethral discharges, fluids from the urethra were collected on calcium alginate swabs. These samples were inoculated in the selective medium Thayer-Martin agar and incubated at 35°C ± 2°C in a 5% CO_2_-enriched atmosphere for 24 h. We obtained four N. gonorrhoeae isolates from different male patients. The colonies were replicated two times on new plates with the same medium. These isolates were identified with the API NH system (bioMérieux SA). Genomic DNA was obtained from isolates using the cetyltrimethylammonium bromide (CTAB) method (7). A 170- to 800-bp normal library was prepared from 500 ng of DNA from each isolate using the Nextera DNA library preparation kit (Illumina). The libraries were sequenced using an Illumina HiSeq 4000 platform, yielding 150-bp paired-end reads. The raw reads were quality controlled and filtered using FastQC v0.11.8 (8) and Trimmomatic v0.36 (9), respectively. The filtered reads were *de novo* assembled and annotated using the Gen2Epi v0.1 pipeline (10). The contigs were scaffolded using a reference genome (GenBank accession number NZ_AP023069.1) with Ragout v2.3 (11) The assembly statistics are shown in Table 1. In the assembled contigs from two isolates (isolates 2312 and 942021), we found plasmids associated with AMR, containing broad-spectrum β-lactamase genes. Isolate 2312 had a pJD4 Asian plasmid (5,724 bp) with the TEM-1 β-lactamase gene *bla*_TEM-P14S_ (12). Isolate 942021 showed the presence of a pSJ5.2 Toronto-type plasmid (5,216 bp) with the *bla*_TEM-135_ allele. These two TEM enzymes have high levels of ampicillin degradation (12). TEM-135-producing isolates have also been associated with high-level ciprofloxacin and tetracycline resistance (13). Additionally, a conjugative plasmid (39,199 bp) lacking the *tet*(M) resistance gene was found in isolates 652 and 942021. The latter has homology (>99.8%) with the plasmids found in the reference strains WHO L, M, O, and W, which still have an unknown role (3). A recent global study demonstrated a high prevalence of AMR plasmids in gonococcal strains isolated in low/middle-income countries (LMICs) and an association with genomic diversity among N. gonorrhoeae strains (14). Here, we found four N. gonorrhoeae isolates with plasmid diversity circulating in a Colombian population subset. This may explain the molecular mechanisms by which we are seeing an increase in AMR, mainly in neglected populations such as homeless communities (15). WGS is a powerful tool for molecular epidemiology studies of N. gonorrhoeae, allowing early detection of AMR and the screening of gonococcal genotypes.

**TABLE 1:** Summary of assembly statistics

Sample	Plasmid(s)	PubMLST sequence type	Genome size (bp)	No. of raw reads	Avg coverage (×)	Contig *N*_50_ (bp)	No. of coding sequences	G+C content (%)	GenBank accession no.	SRA accession no.
2312	pJD1 (cryptic), pJD4 (Asian type), FDAARGOS_205 (conjugative)	8145	2,210,720	57,834,928	3,924	50,012	2,101	52.5	CP097461	SRX15369046
351961	pJD1 (cryptic)	9363	2,242,106	51,557,314	3,449	63,968	2,125	52.4	CP097460	SRX15369047
652	pJD1 (cryptic), WHO_W plasmid 2 (conjugative)	8143	2,174,453	44,623,062	3,078	58,578	2,059	52.7	CP097459	SRX15369048
942021	pJD1 (cryptic), pSJ5.2 (Toronto type), WHO_W plasmid 2	8143	2,177,753	57,400,390	3,953	66,003	2,085	52.7	CP097458	SRX15369049

The project and informed consent forms were evaluated and approved by the Bioethics Committee of the School of Medicine of the University of Antioquia (ethics approval number 2017-022), the Family Advocate from the Colombian Institute for Family Welfare (ICBF), and the Mayor's Office of Medellín, Colombia. Informed consent forms were signed by all participants.

### Data availability.

The whole-genome sequences and raw sequence reads were deposited in DDBJ/ENA/GenBank under the BioProject accession number PRJNA835124.
